# Müllerian adenosarcoma mimicking cervical carcinoma in a 79-year-old woman: diagnostic challenges and laparoscopic treatment: a case report

**DOI:** 10.11604/pamj.2025.52.110.49172

**Published:** 2025-11-13

**Authors:** Fang Zhang, Jie Hu, Jia Bian, Yupeng Lan

**Affiliations:** 1 Gynecology Department, The Affiliated People´s Hospital of Ningbo University, Ningbo, 315000, Zhejiang, PR, China

**Keywords:** Uterine adenosarcoma, cervical neoplasm, uterine tumor, cervical carcinoma, case report

## Abstract

Uterine Müllerian adenosarcoma rarely presents as a cervical “cancer-like” mass; we report the first detailed case in a 79-year-old woman whose 5-cm exophytic lesion mimicked advanced cervical carcinoma on imaging. She presented with 2 weeks of post-menopausal bleeding and a friable polyp protruding through the cervical os; MRI described a bulky cervical tumour extending into the lower uterine segment, while tumour markers remained normal. Initial biopsy was inconclusive; subsequent excision and hysterectomy revealed FIGO stage Ib1 low-grade adenosarcoma originating from the lower uterine segment with superficial cervical involvement. Laparoscopic total hysterectomy with bilateral salpingo-oophorectomy was performed uneventfully, and at 7 months she remains disease-free. The case underlines that adenosarcoma can masquerade as cervical cancer, emphasises the need for generous tissue sampling and WT1/ER-based immunohistochemistry to define tumour origin, and confirms excellent outcomes with complete surgical excision for stage I disease.

## Introduction

Uterine Müllerian adenosarcoma is an uncommon, low-grade biphasic neoplasm that typically arises within the endometrial cavity of perimenopausal or post-menopausal women [1,2]. Primary cervical origin is distinctly rare, accounting for <5% of all cases [3], and the tumour rarely presents as an exophytic “cervical cancer-like” mass. We report a 79-year-old woman whose sole clinical manifestation was recurrent post-menopausal bleeding and a large, friable, 5 cm pedunculated lesion protruding through the cervical os. Pre-operative imaging (pelvic MRI and CT) interpreted the lesion as cervical carcinoma with uterine extension, underscoring the diagnostic challenge. To our knowledge, only a handful of cases of uterine adenosarcoma masquerading macroscopically as advanced cervical cancer have been documented [4,5]. Our case is unique because meticulous step-sectioning and an immunoprofile (WT1, ER/PR, CD10-positive stroma) demonstrated that the tumour actually originated from the lower uterine segment and secondarily involved the cervical mucosa-thereby clarifying a true cervical versus uterine primary. This observation adds to the literature a practical algorithm combining targeted sampling and immunohistochemistry to resolve the site of origin, and supports the safety and efficacy of laparoscopic total hysterectomy with bilateral salpingo-oophorectomy even in octogenarian patients.

## Patient and observation

**Patient information:** a 79-year-old gravida-para post-menopausal woman (last menstrual period 30 years prior) presented to our gynaecology clinic with a 2-week history of painless, recurrent post-menopausal vaginal bleeding associated with malodorous discharge and a self-palpated “rough” vaginal mass. She denied abdominal pain, weight loss, or urinary/bowel symptoms. Her medical history was notable for poorly controlled essential hypertension of 8 years´ duration (intermittent oral antihypertensive therapy); she had no prior pelvic surgery, hormone-replacement therapy, or tamoxifen exposure. There was no family history of gynaecologic malignancy or known hereditary cancer syndrome, and she lived independently without social or financial barriers to care. Previous interventions consisted only of outpatient blood-pressure monitoring and lifestyle advice; no prior gynaecologic imaging or biopsies had been performed.

**Figure 1:**
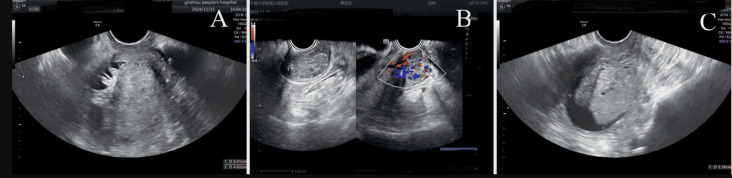
transvaginal ultrasound; A) endometrial intracavitary mass; B) hypoechoic lesion at posterior cervical wall; C) fluid collection within the uterine cavity

**Figure 2:**
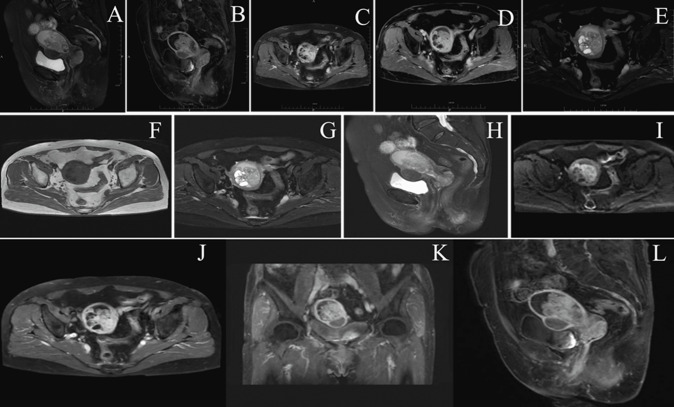
pelvic MRI of adenosarcoma: AB) T1 coronal/sagittal; CD) T2 coronal/sagittal; E) WI; FG) T1 and T2 axial show mixed signal filling uterus; H) T2 sagittal-84 mm cervical mass, uterine involvement, fluid; I) DWI-solid restricted, cystic free; JL) post-contrast solid avid enhancement

**Figure 3:**
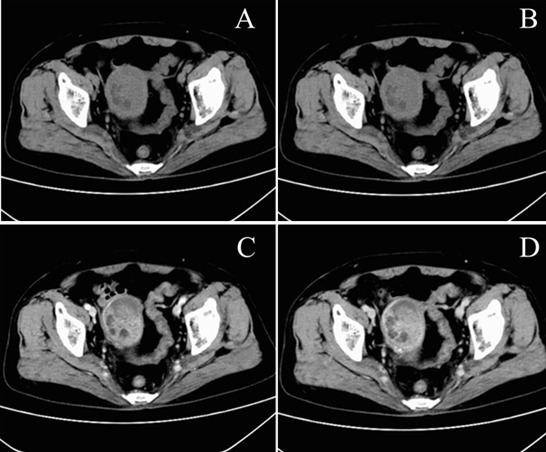
abdominal CT; A) non-contrast: mixed iso-/hypodense uterine mass; B) arterial: minimal enhancement; C) venous: avid solid, absent cystic enhancement; D) delayed: persistent solid, no cystic enhancement

**Clinical findings:** on presentation, vital signs were stable except for hypertension (175/102 mmHg). Pelvic examination revealed a lax vaginal vault with a 56x51x50mm friable, lobulated, polypoid mass protruding through the cervical os and bleeding on contact; the uterus was normal-sized, mobile, and non-tender, with no adnexal masses or parametrial thickening. Transvaginal ultrasound showed an enlarged uterus containing a heterogeneous, highly vascular intracavitary lesion with moderate fluid (Figure 1). Pelvic MRI demonstrated a bulky cervical tumour extending into the lower uterine segment, displaying T1 isointensity, T2 hyperintensity, restricted diffusion, and heterogeneous enhancement; no enlarged lymph nodes were identified (Figure 2). Contrast-enhanced abdominal-pelvic CT corroborated a 55x44mm mixed-density uterine mass and a 27x28mm hyper-enhancing cervical nodule without evidence of distant metastasis (Figure 3). All serum tumour markers (CA-125, SCC-Ag, CEA) remained within normal limits.

**Timeline of the current episode:** weeks before admission-post-menopausal vaginal bleeding begins; day 0-presents to clinic with malodorous discharge and self-palpated mass; day 1-TVUS, pelvic MRI & abdominal-pelvic CT show bulky cervical/lower-uterine lesion suspicious for cervical cancer; day 2-office biopsy reads“cervical polyp with atypical stroma”; day 7-under spinal anaesthesia, the patient underwent complete excision of the cervical mass along with dilation and curettage of the uterine cavity; intra-operative frozen-section analysis indicated a low-grade sarcoma; day 10-permanent pathology confirms uterine adenosarcoma; day 17-laparoscopic total hysterectomy + bilateral salpingo-oophorectomy completed; day 21-discharged home; 7 months later-follow-up confirms no recurrence.

**Diagnostic assessment:** initial diagnostic work-up included pelvic examination, transvaginal ultrasound (heterogeneous 50x46mm vascular intracavitary lesion), pelvic MRI with DWI (bulky cervical/lower-uterine mass with restricted diffusion), and routine tumour markers (all normal); an office punch biopsy yielded scant tissue reported as “cervical polyp with atypical stroma,” highlighting the challenge of limited sampling and the need for larger excision to achieve diagnosis. No financial or cultural barriers were encountered, but the differential remained broad-cervical carcinoma, endometrial carcinoma, and sarcoma. Definitive diagnosis was established after complete excisional biopsy and hysterectomy: FIGO stage Ib1 low-grade Müllerian adenosarcoma with <1/3 myometrial invasion and no lymphovascular or extrauterine involvement, conferring a favourable prognosis with >90% disease-specific survival at 5 years.

**Diagnosis:** FIGO stage Ib1 low-grade Müllerian adenosarcoma of the uterus (arising from the lower uterine segment with superficial myometrial invasion and extension into the cervical canal mucosa, no lymphovascular or extrauterine involvement).

**Figure 4:**
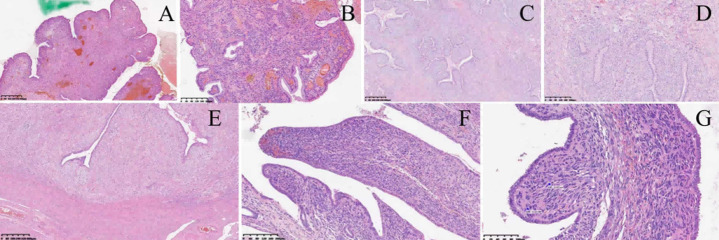
histopathology of cervical/uterine lesions; A) (x40): polypoid mass with benign mucinous glands and hypercellular stroma; B) (x100): periglandular stromal cuffs; C) (x40): mimics phyllodes tumor; D) (x100): periglandular cuffs; E) (x40): infiltrates myometrium; F) (x100): atypical stroma; G) (x200): high-grade stroma with mitoses (arrows)

**Figure 5:**
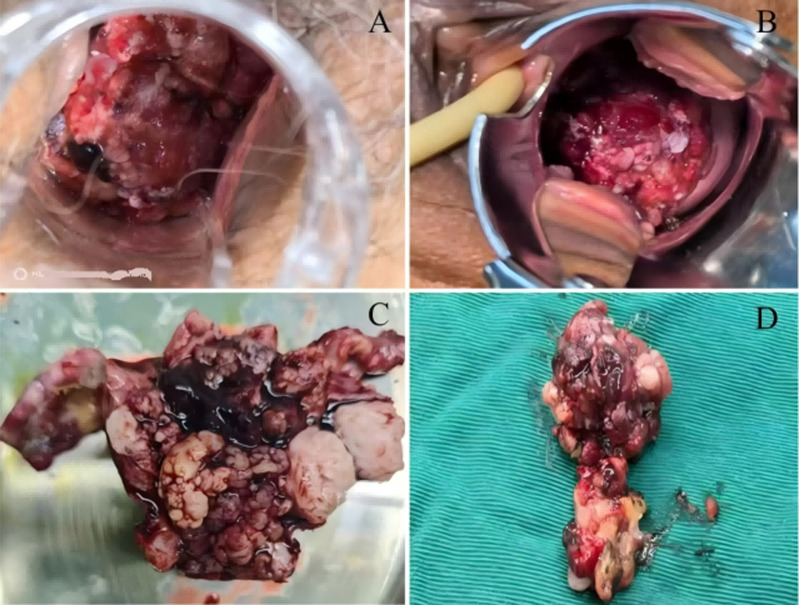
gross examination of surgical specimens: A,B): the vaginal speculum revealed a cauliflower-like, firm mass protruding from the cervical canal; C,D): the removed tumor with necrotic bleeding on the surface

**Therapeutic interventions:** upon admission, a punch biopsy was performed. The histopathological examination of the biopsy specimens (Figure 4 (A,B)) and subsequent expert consultation were insufficient for a definitive diagnosis. However, definitive histopathology from the procedure later established low-grade Müllerian adenosarcoma (Figure 4 (C,D)). Additionally, postoperative assessment of the hysterectomy specimen confirmed FIGO stage Ib1 disease with <1/3 myometrial invasion, negative margins, and no lymphovascular or extrauterine involvement (Figure 4 (E,F,G)). Given these findings, the decision was made to perform a more extensive excisional procedure. On day 7 the patient underwent spinal anaesthesia for complete removal of the 5x5x4cm cervical polypoid mass together with fractional curettage of the endometrium; intra-operative findings confirmed a pedunculated, friable lesion arising at the junction of the upper posterior cervical canal and lower uterine segment, yielding 30 g of grey-yellow tissue (Figure 5). Frozen section at this time suggested low-grade sarcoma. Based on multidisciplinary tumour-board advice, definitive surgical therapy was scheduled. On day 17, a laparoscopic total hysterectomy with bilateral salpingo-oophorectomy, pelvic adhesiolysis and high ligation of the ovarian vessels was performed without intra-operative complications. No adjuvant chemotherapy, radiotherapy or hormonal therapy was administered. Post-operative analgesia consisted of intravenous paracetamol 1 g every 6 h for 48 h followed by oral paracetamol 650 mg as needed, and thromboprophylaxis with enoxaparin 40 mg subcutaneously daily until discharge. No further treatment modifications have been required during 7 months of surveillance.

**Follow-up and outcomes:** post-operative surveillance was conducted every three months in the outpatient clinic and included gynaecological examination, serum CA-125 and SCC-Ag measurements, and pelvic MRI. At the most recent 7-month visit, the patient remained asymptomatic, pelvic examination was unremarkable, and imaging revealed no evidence of local recurrence or distant metastasis; tumour markers remained within the normal range. Adherence to the surveillance protocol was confirmed by telephone questionnaire, and no appointments were missed. No surgical complications (bleeding, infection, or bowel obstruction) or other adverse events were recorded during the follow-up period.

**Patient perspective:** the patient was delighted with the quality of care.

**Informed consent:** written informed consent was obtained from the patient for the publication of this case report.

## Discussion

The principal strength of this report lies in the detailed clinico-radiological description followed by complete surgical staging, allowing unequivocal assignment of FIGO stage Ib1 and confirmation of an unusual site of origin (lower uterine segment with secondary cervical involvement). Step-wise pathological review, including consultation at a tertiary centre and a comprehensive immunohistochemical panel (WT1, ER, PR, CD10, SMA, Ki-67), provides a reproducible model for distinguishing uterine from cervical adenosarcoma when imaging is inconclusive. Limitations include the absence of long-term follow-up beyond seven months and the lack of molecular analysis (e.g., next-generation sequencing) that might further elucidate pathogenesis.

Adenosarcoma accounts for 0.5-2% of all uterine malignancies and <5% of uterine sarcomas, with cervical primaries representing <10% of these cases [1,3]. The typical presentation-post-menopausal bleeding and/or a polypoid mass-is non-specific, and pre-operative diagnosis is achieved in 15% of patients [5]. MRI features such as a bulky, heterogeneous, T2-hyperintense mass with cystic change and restricted diffusion have been described [6]; however, these findings overlap extensively with cervical carcinoma, as highlighted in our case. Histologically, the diagnostic hallmark is a biphasic architecture of benign-appearing glands surrounded by a sarcomatous stroma that often condenses beneath the epithelium (“cambium layer”) [2]. Immunohistochemistry is valuable: WT1, ER and PR are consistently positive in the stromal component, whereas p16 is usually negative, helping to exclude high-grade carcinomas and HPV-related lesions [7]. Treatment is primarily surgical; stage I disease managed by total hysterectomy with bilateral salpingo-oophorectomy is associated with 5-year disease-specific survival rates >90%, and adjuvant therapy is not recommended in the absence of sarcomatous overgrowth or extrauterine spread [8,9].

The observation that a lower uterine segment adenosarcoma can manifest as a cervical “grape-like” mass underscores the importance of generous tissue sampling and expert pathology review. In our patient, the absence of deep myometrial invasion, vascular invasion or sarcomatous overgrowth predicted a favourable prognosis and justified a conservative post-operative approach. The rapid laparoscopic completion surgery in an octogenarian also corroborates recent data demonstrating the feasibility of minimally invasive techniques in this age group without compromising oncological safety [1]. Clinicians should consider adenosarcoma in any post-menopausal woman presenting with bleeding and a polypoid cervical mass, even when imaging suggests cervical carcinoma. Adequate tissue sampling, WT1/ER/PR immunohistochemistry, and central pathology review are critical to establish the diagnosis and to distinguish uterine from cervical origin. Early complete surgical excision offers excellent outcomes, and routine adjuvant therapy is unnecessary for stage I low-grade disease.

## Conclusion

This case illustrates that Müllerian adenosarcoma can masquerade as cervical carcinoma, both clinically and radiologically, especially in post-menopausal women presenting with bleeding and an exophytic cervical mass. Accurate diagnosis hinges on generous tissue sampling, expert pathological review and targeted immunohistochemistry; once established, complete laparoscopic hysterectomy with bilateral salpingo-oophorectomy provides excellent oncological control for stage Ib1 low-grade disease without the need for adjuvant therapy. The key lesson is to maintain a high index of suspicion for adenosarcoma whenever a bulky cervical lesion yields atypical histology, ensuring timely referral and curative treatment even in elderly patients.
